# A Convex Optimization Approach to Multi-Robot Task Allocation and Path Planning

**DOI:** 10.3390/s23115103

**Published:** 2023-05-26

**Authors:** Tingjun Lei, Pradeep Chintam, Chaomin Luo, Lantao Liu, Gene Eu Jan

**Affiliations:** 1 Department of Electrical and Computer Engineering, Mississippi State University, Mississippi State, MS 39762, USA; 2 Department of Intelligent Systems Engineering, Indiana University, Bloomington, IN 47408, USA; 3 Department of Electrical Engineering, National Taipei University, New Taipei City 23741, Taiwan; 4 Tainan National University of the Arts, Tainan City 72045, Taiwan

**Keywords:** multi-robot deployment, convex optimization, task allocation, SOM neural networks, path planning, task decomposition

## Abstract

In real-world applications, multiple robots need to be dynamically deployed to their appropriate locations as teams while the distance cost between robots and goals is minimized, which is known to be an NP-hard problem. In this paper, a new framework of team-based multi-robot task allocation and path planning is developed for robot exploration missions through a convex optimization-based distance optimal model. A new distance optimal model is proposed to minimize the traveled distance between robots and their goals. The proposed framework fuses task decomposition, allocation, local sub-task allocation, and path planning. To begin, multiple robots are firstly divided and clustered into a variety of teams considering interrelation and dependencies of robots, and task decomposition. Secondly, the teams with various arbitrary shape enclosing intercorrelative robots are approximated and relaxed into circles, which are mathematically formulated to convex optimization problems to minimize the distance between teams, as well as between a robot and their goals. Once the robot teams are deployed into their appropriate locations, the robot locations are further refined by a graph-based Delaunay triangulation method. Thirdly, in the team, a self-organizing map-based neural network (SOMNN) paradigm is developed to complete the dynamical sub-task allocation and path planning, in which the robots are dynamically assigned to their nearby goals locally. Simulation and comparison studies demonstrate the proposed hybrid multi-robot task allocation and path planning framework is effective and efficient.

## 1. Introduction

Multi-robot deployment is an essential issue in robotics field, which requires mobile robots to be deployed in the workspace to cooperatively fulfill tasks [1,2,3,4,5]. Dynamic deployment problems in combinatorial optimization consist of finding an optimal solution in some objectives, such as timing, workload, distance traveled, energy, and deadlines [6,7,8,9,10,11,12,13]. In real-world applications, such as mine detection, environmental exploration, and rescue mission, many robots need to be dynamically deployed to goals (targets) while total distance cost among robots, and between robots and goals is minimized [14,15,16,17,18,19,20].

### 1.1. Related Works

Although there have been many studies on multi-robot deployment via optimization models, very few existing deployment algorithms focus on distance constraints. Previous research on multi-robot deployment and task allocation may be categorized into various methods, such as distributed-based [21,22,23,24], linear programming [24,25], graph-based [26,27,28], market-based [22,29,30], conflict-based search method [31], neural networks [32,33], etc. Sung et al. [21] addressed a distributed approach to integrate multi-robot deployment and multi-target tracking. A local algorithm is adopted to achieving performance close to the optimal algorithms with limited communication with assistance of distributed approach. For a heterogeneous multi-robot system, where tasks form disjoint groups and where there are restrictions on the number of tasks, a robot may accomplish (both within the overall mission and within each task group), Luo et al. [34] provided a provably-good distributed task allocation methods. Their goal of task allocation is to maximize (minimize) the total payout (cost) of the robots, whereby each robot receives a payment (or incurs a cost) for completing a job. Basil et al. [35] proposed a modular robot to be a morphologically flexible, autonomous kinematic machines with hundreds or even millions of modules that work together to exhibit intelligent behavior. The self-reconfiguration process, which seeks to identify a series of reconfiguration activities to convert robots from an initial form to a target one, may be enhanced by clustering the modules in modular robots. Luo et al. [24] extended distributed algorithms by taking deadline into account in multi-robot deployment. Purohit et al. [26] presented a spanning tree method associated with self-localizing capability in the graph. However, robot teams are not yet successfully clustered and assigned to multiple tasks effectively. To efficiently and securely explore and recon a given region with a large number of robots, Li et al. [36] introduced an enhanced genetic algorithm (IGA) to tackle the job assignment issue of a multi-robot system. Searching the numerous identical sections of the specified region is a subtask that must be completed in order to solve a challenge. Qin et al. [37] formally described the challenge of completing dynamic tasks with several robots by modeling their interactions as a series of state transitions, or behavior trees. Through the use of a unique priority system, a framework-associated distributed algorithms for inter-robot communication, negotiation, and agreement protocols was provided. Bai et al. [38] investigated the multi-robot task allocation issue, in which a team of geographically-dispersed robots must effectively move a number of packages from their originating locations to their respective destinations within a certain amount of time. A market-based approach is suggested by Rossi et al. [29] for simultaneous task subdivision and allocation in heterogeneous multi-robot systems. García et al. [30] implemented a behavior-based architecture with many layers allowing the market-based method to achieve varying degrees of coordination. However, as the number of robots in the team or the complexity of the problem increases, market-based approaches suffer from scalability and dynamics issues, which tend to hinder these processes, especially when this happens in real time.

Neural network (NN) methods have been broadly applied to multi-robot path planning and task allocation. Luo and Yang [33] developed a neural dynamics model to assign multiple robots to environmental exploration collaboratively. Multiple robots cooperate to achieve a common sweeping goal effectively. However, energy consumption of multi-robot system has not been considered in this paper. Later, Luo et al. [32] extended their research by addressing the computational complexity and energy efficiency of multi-robot system with navigation [39]. The energy consumption of multiple tasks for an arbitrary number of robots is considered, in which a bio-inspired neural networks model for multi-robot navigation applied to cleaning robots is developed. In this model, multiple robots are assigned to complete terrain coverage task cooperatively, extendable to unknown exploration environments [39]. Distance cost optimization is considered in the recent research. For instance, Lee et al. [31] suggested a master–slave based multi-robot deployment with time and distance minimization consideration; however, the distance is not globally optimized. Some researchers combined a couple of models to take advantage of various benefits. For instance, Michael et al. [22] effectively integrated distributed algorithm and market-based method for multi-robot deployment. Motes et al. [40] concatenated path-finding method and conflict-based search method to multi-robot deployment and navigation with inter-team conflict avoidance.

The centralization or decentralization of task allocation methods is another essential characteristic. Using the well-known Kiva warehouse robot as an example [41], both task allocation and path planning are conducted centrally; however, this may not be feasible if the multi-robot system operates in an environment that lacks robust sensing and computational capabilities. Clustering is a potential middle alternative between centralized and decentralized methods that aims to balance performance and computational load [42]. Martin et al. [43] proposed an algorithm to group the players into balanced clusters, applied randomized methods to large problems to relieve the computational load, and assessed feasibility in a large scenario and contrasted with a genetic approach.

In general, market/auction-based approaches can be solved decentrally [44]. However, the problem structure must be straightforward enough so that each agent can act as a bidder and bid on tasks; this can be challenging when some tasks require the cooperation of multiple agents. Optimization-based methods permit more complex problem structures but can be challenging to solve decentralized. Bo et al. [45] proposed a stochastic programming framework, which optimizes the decomposition, allocation, and scheduling of tasks for a group of agents. The framework enables teams of mobile robots in different locations to perform different tasks. However, the scalability and dynamics issues arise as the number of robots in the team or the complexity of the problem increases. Therefore, a framework based on optimization is proposed in this paper for decentralized task allocation that is appropriate for complex problem models.

### 1.2. Proposed Framework and Original Contributions

**Figure 1:**
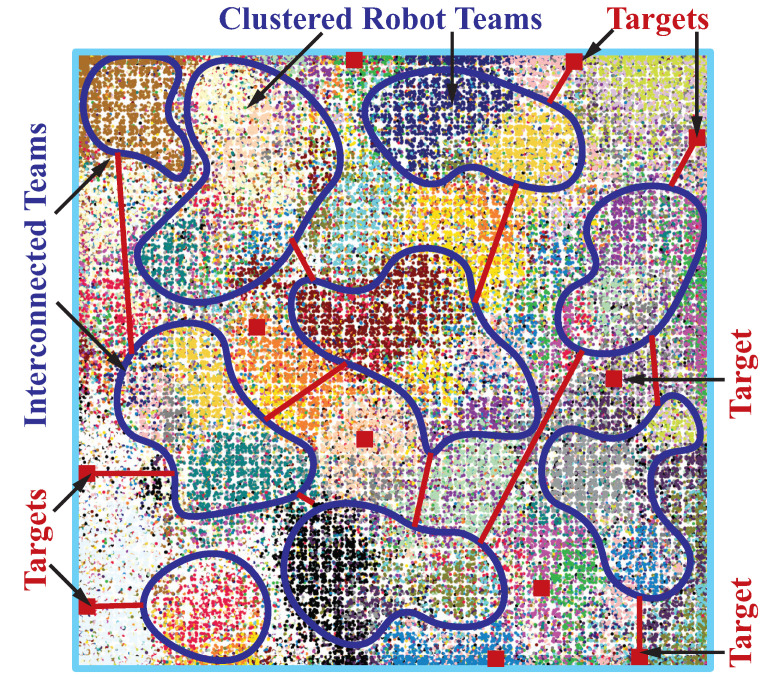
Illustration of an exploration environment with robot teams (swarms) (adapted from Eklavya 2019 [46]). In this multi-robot deployment problem, a *known* environment is given with N target locations, in which some locations are located within the workspace (such as warehouses), but other locations are in the edges of the workspace. There are varying numbers of robots in each team.

In this paper, a two-stage team-based decentralized deployment framework through convex optimization model is developed. It aims for multiple robots scattered in arbitrary space to minimize distance cost among robots, and between robots and goals so as to reach all the task locations effectively shown in Figure 1.

In the first stage, multiple robots in workspace are classified and clustered into a variety of teams enclosing correlative robots as *task decomposition*. Robot teams in arbitrary shape are represented as circles, in order to formulate it into a convex optimization model. The relative locations of teams are obtained through this convex optimization model. Then, the locations of circles are refined in a refinement stage via a Delaunay triangulation method to clean up the overlaps of teams. In the second stage, within the team, a self-organizing map (SOM) neural networks (NN) paradigm is considered to complete the dynamical sub-task allocation and path planning by dynamically assigning them to their nearby goals locally. The proposed framework couples task decomposition, deployment, local sub-task allocation and path planning to support cases where the optimal solution depends on robot interrelation, dependencies, and availability, as well as inter-team conflict avoidance.

The contributions of this paper are summarized as follows:

(1) A two-stage convex-optimization-based framework is proposed for decentralized multi-robot task allocation and task decomposition.
(2) The first stage of the proposed convex-optimization-based framework is designed to determine the relative locations of the robot teams. By obtaining data regarding the robots and the targets, the robots are categorized and aggregated into multiple teams with respect to the total distance cost.
(3) The second stage of the proposed convex optimization-based framework aims to locally assign teams of robots to final goals. The local self-organizing map based neural network (SOMNN) method is developed to subtask allocations and robot path planning.
(4) A Delaunay triangulation (DT) method is employed to refine the team locations and, thus, connect the two stages.

**Figure 2:**
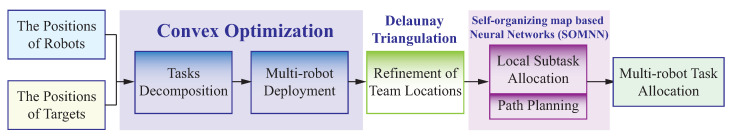
The overall workflow of team-based decentralized deployment framework through convex optimization models.

The overall workflow of the proposed multi-robot task allocation framework is illustrated in Figure 2.

The rest of this paper is organized as follows. In Section 2, the problem statement and formulation is presented. In Section 3, the distance optimal-based convex optimization model for multi-robot deployment is proposed. Section 4 presents the SOMNN sub-task allocation and path planning approach. Numerical experiments, simulations, and comparison studies are presented in Section 5. Several important properties of the presented framework are summarized in Section 6.

## 2. Problem Statement and Formulation

**Figure 3:**
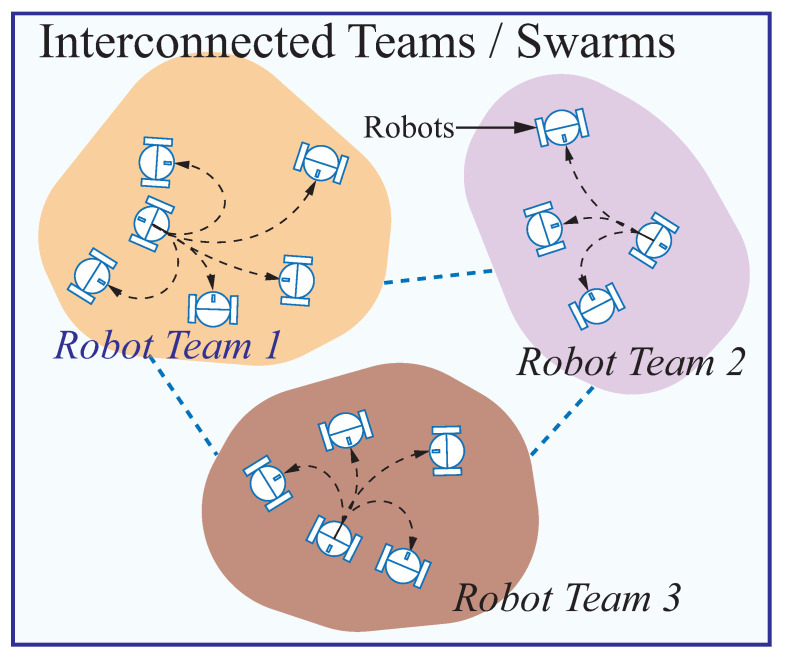
Illustration of multi-robot deployment with robot teams. Robots move as a swarm while minimizing the total distance cost between them. The classification of interrelated robots into the relevant swarm for deployment on adjacent terrain. Some robots are contained within one swarm in conjunction with robots from other swarms.

In this multi-robot deployment problem, a known environment is given with N target locations, in which some locations are located within the workspace (such as warehouses), but other locations are in the edges of the workspace (see Figure 1). There are *m* robots to be deployed that are classified into *g* swarms (groups). In every swarm, there are *k* robots (*k* is a variable). Robots are initially located within different irregular shapes as swarms (see Figure 1). Robots moves as a swarm while maintaining a minimized total distance cost among robots shown in Figure 3. Interrelated robots are classified into the same swarm to be deployed in a nearby terrain. Some robots are enclosed in one swarm in collaboration with the robots in other swarms. Robots with relatively high connections are arranged close to one another for connectivity. The distance between pairs of robots with high connection is to be minimized. Likewise, the distance between robots and target locations is to be minimized.

The relative locations of the robot swarms in the known environment are provided by an attractor–repeller convex optimization algorithm. The robots enclosed in swarms are deployed into their relative locations in the workspace. Given the relative locations of the robots, a planar graph and relative location matrix are obtained by a Delaunay triangulation approach, to enforce no overlap between any two circles, used for the next step. The robots contained in their swarms are locally assigned to their locations. The robots in a group are required to have the shortest possible distance from the fixed target location on the edge of the working environment.

## 3. Convex Optimization Model

The proposed framework couples task decomposition, deployment, local subtask allocation and path planning to support scenarios where the optimal solution depends on robot interrelation, dependencies, and availability, as well as inter-team conflict avoidance. We introduce an efficient technique that addresses the deployment problem of a team of heterogeneous robots. For multiple scattered tasks in arbitrary space, the objective to be solved is to minimize distance for robots to reach all the task locations.

### 3.1. Convex Optimization Algorithms

Convex optimization algorithms to minimize the total distance cost are described in this section. There are several definitions of the proposed algorithms.

**Definition** **1.** *Team of robots.*

The deployment of robot swarms is described as follows:

- Each swarm of robots is labeled 1, 2, ..., *M*, represented as a circle with radius $r_{i}$, *i* = 1, 2, ..., *M*. The radius $r_{i}$ is determined by the number of robots in this swarm.
- The location of each swarm 1, 2, ..., *M* is given by the coordinates of its center depicted as ($x_{i}$, $y_{i}$).
- The non-negative cost per unit distance between swarms *i* and *j* is denoted by $c_{ij}$, which is equivalent to the weight between swarms.
- The distance measured from center to center of swarms *i* and *j* by Euclidean distance ($L_{2}$ norm) is represented by $d_{ij}$, that is, $d_{ij}=\sqrt{{\left(x_{i}-x_{j}\right)}^{2}+{\left(y_{i}-y_{j}\right)}^{2}}$.

Therefore, the multi-robot deployment problem may be mathematically formulated as weighted distance minimization model among swarms as follows.

(1) $$\begin{array}{cc} \; & \min_{\left(x_{i},y_{j}\right)}\;\sum_{1\leq i<j\leq N}c_{ij}d_{ij}\\ \; & s.t.\;\;r_{i}+r_{j}-d_{ij}\leq 0,\;\forall \;1\leq i<j\leq M \end{array}$$

The circles containing robots are deployed as close as possible, but they should not overlap. The objective function $\sum_{1\leq i<j\leq M}c_{ij}d_{ij}$ attempts to make distance $d_{ij}$ as low as possible, which attracts pairs of circles *i* and *j* towards each other so as to function as an attractor. The constraints, $r_{i}+r_{j}\leq d_{ij}\left(\forall \;1\leq i<j\leq M\right)$, push any pair of circles away from each other with no overlapping [47]. Swarms are approximated by circles whose radii are proportional to the amount of encircled robots. The constraint to prevent circles from overlapping has the mathematical form: ${\left(x_{i}-x_{j}\right)}^{2}+{\left(y_{i}-y_{j}\right)}^{2}\geq {\left(r_{i}+r_{j}\right)}^{2},$ where $\left(x_{i},y_{i}\right)$ and $\left(x_{j},y_{j}\right)$ denote the coordinates of the centers of two circles *i* and *j*, whereas $r_{i}$ and $r_{j}$ represent their corresponding radii, respectively. The distance between circles is minimized, while they have no overlaps as constraints. This problem may be formulated as a non-linear optimization model with its superiority of convexity, inspired by a dynamic spring system [48] as follows.

(2) $$\begin{array}{cc} \; & \min_{\left(x_{i},y_{j}\right),w_{F},h_{F}}\;\sum_{i,j\in M}\frac{1}{2}c_{ij}d_{ij}^{2}\;+\sum_{i,j\in M}\max\left\{0,\omega_{ij}\left(r_{i}+r_{j}-d_{ij}\right)\right\}\\ \; & s.t.\\ \; & \;\;\frac{1}{2}w_{F}\geq x_{i}+r_{i}\;\;\mathrm{and}\;\;\frac{1}{2}w_{F}\geq r_{i}-x_{i},\;\mathrm{for}\;\mathrm{all}\;i\in M, \end{array}$$

(3) $$\begin{array}{cc} \; & \;\;\frac{1}{2}h_{F}\geq y_{i}+r_{i}\;\;\mathrm{and}\;\;\frac{1}{2}h_{F}\;\geq r_{i}-y_{i},\;\;\mathrm{for}\;\mathrm{all}\;i\in M,\\ \; & \;\;w_{F}^{up}\geq w_{F}\geq w_{F}^{low},\\ \; & \;\;h_{F}^{up}\;\geq h_{F}\;\geq h_{F}^{low}, \end{array}$$

where $\omega_{ij}$ is a constant, $\omega_{ij}>0$. $\omega_{ij}\left(r_{i}+r_{j}-d_{ij}\right)$ is penalized in the total energy function to generate a repulsive force. $d_{ij}$ and $r_{i}$, $r_{j}$ of circles are defined above. If $d_{ij}<r_{i}+r_{j}$, then circles *i* and *j* overlap. $d_{ij}\geq r_{i}+r_{j}$ prevents circles *i* and *j* from overlapping each other.

In order to formulate convex optimization model, in which circles enclosing robots are formulated to have no overlaps, repulsive force is introduced. In addition to this attractive force, we consider move this max function by introducing repulsive force. Afterwards, robots as swarms are deployed into their appropriate potions. Target distance concept employed in this model was initially introduced by Anjos and Vannelli [49]. Let each swarm of robots *i* be represented by a circle with radius $r_{i}$, where $r_{i}$ is proportional to $\sqrt{a_{i}}$, the square root of the area of circle *i*, measured by the amount of robots encircled. Following [49], the target distance for each pair of circles i,j is defined as $t_{ij}≔\alpha {\left(r_{i}+r_{j}\right)}^{2}$, where α>0 is a parameter. To prevent circles from overlapping, the target distance is enforced by introducing a penalty term $f(\frac{D_{ij}}{t_{ij}})$, where $f\left(z\right)=\frac{1}{z}-1$ for z>0, and $D_{ij}={\left(x_{i}-x_{j}\right)}^{2}+{\left(y_{i}-y_{j}\right)}^{2}$. The objective function is thus given by

(4) $$\sum_{1\leq i<j\leq n}c_{ij}D_{ij}+f\left(\frac{D_{ij}}{t_{ij}}\right).$$

The repeller term (i.e., the penalty function) enforced by holding a positive value, functions as a repulsive force, if $r_{i}+r_{j}>d_{ij}$, to hinder the circles from overlapping.

**Figure 4:**
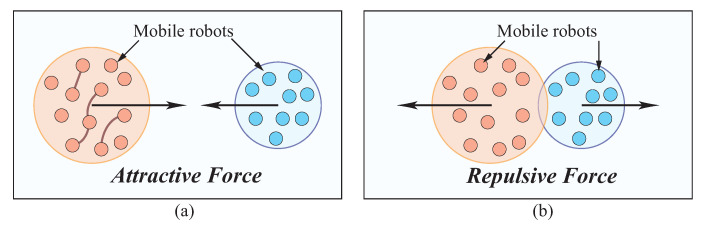
Two circles with attractive and repulsive forces. (**a**) Two disconnected circles with attractive force, $r_{i}+r_{j}\leq d_{ij}$; (**b**) Two connected circles with repulsive force, $r_{i}+r_{j}>d_{ij}$.

The attractor in the objective function used to apply an attractive force to the two circles, makes the two swarms of robots (circles) move closer together (see Figure 4). The repeller term disappears or becomes a negative value implying that there is no any overlap between circles, if $r_{i}+r_{j}\leq d_{ij}$. An attractive force is enforced to the two circles through the attractor in the objective function enforces, if $D_{ij}\geq t_{ij}$. In this case, there is no any overlap between circles and the repeller term is zero or slightly negative. Conversely, the repeller term is positive, which tends to positive infinity as $D_{ij}$ tends to zero so as to prevent the circles from overlapping fully, if $D_{ij}<t_{ij}$. There is no force between circles *i* and *j* exactly if $D_{ij}^{2}=t_{ij}/c_{ij}$. The generalized target distance $T_{ij}$ is defined to contribute to this optimization model.

The model aims to ensure that $\frac{D_{ij}}{t_{ij}}=1$ at optimality, so choosing α<1 sets a target value $t_{ij}$ that allows some overlap of the respective circles, which means that the non-overlap requirement is relaxed. In practice, by properly adjusting the value of α we achieve a reasonable separation between all pairs of circles. Let *M* and *P* denote the set of mobile teams (circles) and the set of targets (goals), respectively. Target distances are applied only for pairs of mobile robot teams (circles). The complete attractor-repeller (AR) model is

(5) $$\begin{array}{cc} \; & \min_{\left(x_{i},y_{i}\right),i\in M,w_{F},h_{F}}\sum_{i,j\in M\cup P}c_{ij}D_{ij}+\sum_{i,j\in M}f\left(\frac{D_{ij}}{t_{ij}}\right)\\ \; & s.t.\\ \; & \;\;x_{i}+r_{i}\leq \frac{1}{2}w_{F}\;\;\mathrm{and}\;r_{i}-x_{i}\leq \frac{1}{2}w_{F},\;\mathrm{for}\;\mathrm{all}\;i\in M,\\ \; & \;\;y_{i}+r_{i}\leq \frac{1}{2}h_{F}\;\;\mathrm{and}\;r_{i}-y_{i}\leq \frac{1}{2}h_{F},\;\;\mathrm{for}\;\mathrm{all}\;i\in M,\\ \; & \;\;w_{F}^{low}\leq w_{F}\leq w_{F}^{up},\\ \; & \;\;h_{F}^{low}\;\leq h_{F}\;\leq h_{F}^{up}, \end{array}$$

where ($x_{i}$, $y_{i}$) are the coordinates of the centre of circle *i* as previously defined; $w_{F}$, $h_{F}$ are the width and height of the workspace; and $w_{F}^{low}$, $w_{F}^{up}$, $h_{F}^{low}$, and $h_{F}^{up}$ are the lower and upper bounds on the width and height, respectively. The first two sets of constraints require that all the circles be entirely contained in the workspace, and the remaining two pairs of inequalities bound the width and height of the workspace of robots. In particular, for certain robot environment, we set $w_{F}^{low}=w_{F}^{up}={\bar{w}}_{F}$ and $h_{F}^{low}=h_{F}^{up}={\bar{h}}_{F}$, where ${\bar{w}}_{F}$ and ${\bar{h}}_{F}$ are the width and height of the workspace to be explored by robots.

**Definition** **2.** *Generalized target distance*

(6) $$\begin{array}{c} T_{ij}≔\sqrt{\frac{t_{ij}}{c_{ij}+\varepsilon }}, \end{array}$$

*where ε>0 is sufficiently small to ensure that $D_{ij}\approx \sqrt{\frac{t_{ij}}{c_{ij}}}$ if $D_{ij}\approx T_{ij}$.*

In real-world applications, the distances $D_{ij}$ between the circles should be inversely proportional to $c_{ij}$ representing the weights on the wire-length, and should be proportional to the relative size of the teams through the value of $t_{ij}$. Hence, a generalized target distance, $T_{ij}$, is defined such that $D_{ij}\approx T_{ij}$ at optimality. Using $T_{ij}$, a convex version of the AR model may be described in the following section with the following term.

(7) $$F_{ij}\left(x_{i},x_{j},y_{i},y_{j}\right)≔\left\{\begin{array}{cc} c_{ij}z+\frac{t_{ij}}{z}-1, & z\geq T_{ij}\;\\ 2\sqrt{c_{ij}t_{ij}}-1, & 0\leq z<T_{ij} \end{array}\right.$$

with $z={\left(x_{i}-x_{j}\right)}^{2}+{\left(y_{i}-y_{j}\right)}^{2}$. It is clear that this problem is convex, and that by construction $F_{ij}$ attains its minimum value whenever the locations of circles *i* and *j* satisfy $D_{ij}\leq T_{ij}$. This includes the case where $D_{ij}=0$, i.e., both circles completely overlap. The idea is to add to the objective function a term of the form $-\ln\left(D_{ij}/T_{ij}\right)$ for each pair i,j of circles. Hence, the model solved in the first stage of our method is

(8) $$\begin{array}{cc} \; & \min_{\left(x_{i},y_{j}\right),w_{F},h_{F}}\sum_{1\leq i<j\leq n}F_{ij}\left(x_{i},x_{j},y_{i},y_{j}\right)-\beta K\ln\left(\frac{D_{ij}}{T_{ij}}\right)\\ \; & s.t.\\ \; & \;\;x_{i}+r_{i}\leq \frac{1}{2}w_{F}\;\;\mathrm{and}\;r_{i}-x_{i}\leq \frac{1}{2}w_{F},\;\mathrm{for}\;\mathrm{all}\;\mathrm{modules}\;i,\\ \; & \;\;y_{i}+r_{i}\leq \frac{1}{2}h_{F}\;\;\mathrm{and}\;r_{i}-y_{i}\leq \frac{1}{2}h_{F},\;\;\mathrm{for}\;\mathrm{all}\;\mathrm{modules}\;i,\\ \; & \;\;w_{F}^{low}\leq w_{F}\leq w_{F}^{up},\\ \; & \;\;h_{F}^{low}\;\leq h_{F}\;\leq h_{F}^{up}, \end{array}$$

where β is a parameter selected empirically. *K* is chosen to reflect the weights of all the pairs of mobile circles (teams) in the objective function by $K=\sum_{i<j}c{}_{ij}$.

**Figure 5:**
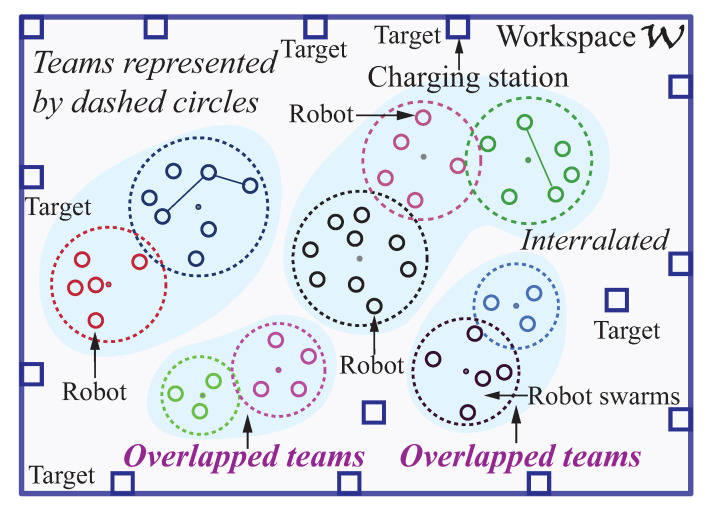
Robot teams (swarms) deployment initially resolved by the proposed convex optimization model.

The topological relationships between terms are obtained in this first stage. Without the term $-\beta K\ln\left(D_{ij}/T_{ij}\right)$ in Equation (8), this problem is convex. By solving it, the solution of the first stage provides relative locations within the workspace for all the robot teams (circles) represented by circles. The relative locations of the robot swarms in the known environment are provided by an attractor–repeller convex optimization algorithm shown in Figure 5. The team-based convex optimization algorithm for robot deployment is summarized in Algorithm 1.
**Algorithm 1:** Team-based convex optimization algorithm for robot deployment**Input:** Initial configuration ($q_{s}$), Goal configuration ($q_{g}$), and Required cost $c_{ij}$, $d_{ij}$**Output: **Path (or sequence of nodes from $q_{s}$ to $q_{g}$), Robots’ final locations ($L_{g}oal$)**Begin**1: Classify *m* robots into *k* teams $T_{i}$, i∈[1,k];2: Approximate teams $T_{i}$ to *k* circles at center of $T_{j}\left(x_{j},y_{j}\right)$, j∈[1,k];3: Solve the convex optimization model (Equation (8)) having $C_{j}\left(x_{j},y_{j}\right)$, j∈[1,k];4: Obtain relative locations of teams. New circle locations ${\tilde{C}}_{j}\left({\tilde{x}}_{j},{\tilde{y}}_{j}\right)$, j∈[1,k];5: Apply SOMNN to subtask allocation to reach *g* goals $G(x_{l},y_{l})$, l∈[1,g];**End**

### 3.2. Refinement of Team Locations

**Figure 6:**
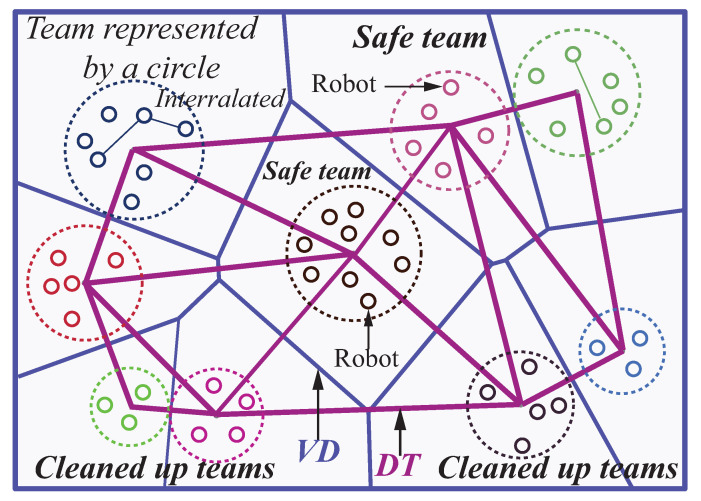
Delaunay triangulation used to linearize and refine the overlapped circles/teams.

The circles are allowed to overlap in the convex optimization stage and the precise locations of team have not been determined. The solution of the first stage provides relative locations for all the circles enclosing robots. In this paper, we consider a relative location matrix to encode relative locations. Using this technique the non-overlap constraints that are originally disjunctive, non-linear, and non-convex can be linearized and easily enforced in the second stage model. We consider the Delaunay triangulation (DT) method for two main reasons, (i) it spreads out circles thus teams in the workspace and (ii) it transforms the relative location graph into a planar graph. In this framework, robot deployment resolved by the convex optimization with relative locations in Figure 5 is refined by the proposed DT method shown in Figure 6.

## 4. SOM Neural Networks for Sub-Task Allocation and Path Planning

**Figure 7:**
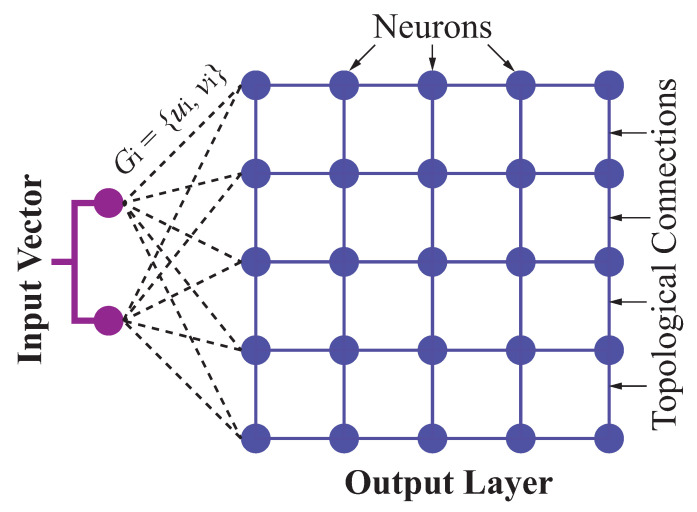
SOM-based NN architecture used for subtask allocation.

In this stage, a swarm of robots are assigned locally as sub-tasks to their goals through self-organizing map based neural networks (SOMNN) method in static or dynamic environments. The principal technical superiority of this SOMNN model is well established, given that the robot trajectory planning is fused with the sub-task allocation in every team and their goals. Once the relative locations of teams with robots are defined, the robots move to their goals in the dynamical environments. In our research, there are *k* teams, each of which has a swarm of robots. There are α robots in one team $T_{1}\left(\alpha \right)$ in a workspace, which are assigned to η goals with pre-defined locations, such as recharging pile for autonomous electric vehicles. In this paper, SOM-based neural network model is made up of two layers of neurons (nodes). The first layer configured as the input layer consists of two neurons ($u_{i},v_{i}$) representing coordinates of the *i*th goal $G_{i}\left(u_{i},v_{i}\right)$. The second layer as the output layer contains α×η neurons, denoted as $r_{1}^{1},r_{1}^{2},\ldots ,r_{1}^{\eta },r_{2}^{1},r_{2}^{2},\ldots ,r_{2}^{\eta },r_{\alpha }^{1},r_{\alpha }^{2},\ldots ,r_{\alpha }^{\eta }$, representing the locations of the α robots and their trajectories. All locations of goals form input dataset. Each neuron in the output layer is fully connected to the neurons in the input layer [50] (see Figure 7).

(9) $$\begin{array}{c} N_{\dot{\alpha }}^{\dot{\eta }}⇐={\dot{D}}_{\dot{\alpha }}^{\dot{\eta }}=\min{\left\{D_{\alpha }^{\eta }\right|}_{i},i=1,\ldots ,\alpha ;\dot{\alpha }=1,\ldots ,\alpha \\ \dot{\eta }=1,\ldots ,\eta ;\;\mathrm{and}\;\left\{\dot{\alpha },\dot{\eta }\right\}\in \varpi \} \end{array}$$

(10) $$D_{\alpha }^{\eta }{|}_{i}=D\left(G_{i},r_{\dot{\alpha }}^{\dot{\eta }}\right)\left(1+P\right)$$

(11) $$\;D\left(G_{i},r_{\dot{\alpha }}^{\dot{\eta }}\right)\;\;=\;\;\left|G_{i}-r_{\dot{\alpha }}^{\dot{\eta }}\right|\;\;=\;\;\sqrt{{\left(x_{i}-w_{\dot{\alpha }}^{\dot{\eta }}\left(x\right)\right)}^{2}\;\;+\;\;{\left(y_{i}-w_{\dot{\alpha }}^{\dot{\eta }}\left(y\right)\right)}^{2}}$$

A weight vector connecting the two input nodes to output nodes is defined as $r_{\dot{\alpha }}^{\dot{\eta }}=\langle w_{\dot{\alpha }}^{\dot{\eta }}\left(x\right),w_{\dot{\alpha }}^{\dot{\eta }}\left(y\right)\rangle$, where $\dot{\alpha }=1,2,\cdots ,\alpha$; $\dot{\eta }=1,2,\cdots ,\eta$. The weight vectors of the neurons for each robot are initialized based on the coordinates of the initial robot location. Therefore, α robots move to the η goals while following planned path, due to this SOM neural network algorithm. A winner $N_{\dot{\alpha }}^{\dot{\eta }}$ is determined in light of the following criterion.

(12) $$\begin{array}{c} \;N_{\dot{\alpha }}^{\dot{\eta }}⟸{\dot{D}}_{\dot{\alpha }}^{\dot{\eta }}=\min\left\{D_{\alpha }^{\eta }{|}_{i},i=1,\ldots ,\alpha ;\dot{\alpha }=1,\ldots ,\alpha \right.\\ \dot{\eta }=1,\ldots ,\eta ;\;\mathrm{and}\;\left\{\dot{\alpha },\dot{\eta }\right\}\in \varpi \}, \end{array}$$

(13) $$D_{\alpha }^{\eta }{|}_{i}=D\left(G_{i},r_{\dot{\alpha }}^{\dot{\eta }}\right)\left(1+P\right),$$

where $N_{\dot{\alpha }}^{\dot{\eta }}$ is the $\dot{\alpha }$-th neuron of the $\dot{\eta }$ th in the group of goals. ϖ denotes the set of output neurons on the $\dot{\eta }$ th in the group of goals, which has not yet been a winner. The weighted distance, ${\dot{D}}_{\dot{\alpha }}^{\dot{\eta }}$, is minimum of $D_{\alpha }^{\eta }{|}_{i}$ described as follows.

(14) $$\;D\left(G_{i},r_{\dot{\alpha }}^{\dot{\eta }}\right)\;\;=\;\;\left|G_{i}-r_{\dot{\alpha }}^{\dot{\eta }}\right|\;\;=\;\;\sqrt{{\left(x_{i}-w_{\dot{\alpha }}^{\dot{\eta }}\left(x\right)\right)}^{2}\;\;\;+\;\;{\left(y_{i}-w_{\dot{\alpha }}^{\dot{\eta }}\left(y\right)\right)}^{2}}$$

where P is a parameter determining the equitable distribution of sub-task of workload for robots. $\left|\cdot \right|$ represents the Euclidean distance. The parameter P is expressed as

(15) $$P=\frac{\ell_{\dot{\alpha }}-{\tilde{V}}_{A}}{1+{\tilde{V}}_{A}},$$

where $\ell_{\dot{\alpha }}$ is the trajectory length of the $\dot{\alpha }$ th robot ($\dot{\alpha }=1,2,\cdots ,\alpha$). ${\tilde{V}}_{A}$ is the average trajectory length of the robots, given by the following Equation (16).

(16) $${\tilde{V}}_{A}=\frac{1}{\alpha }\sum_{\dot{\alpha }=1}^{\alpha }\ell_{\dot{\alpha }}$$

The winner implies the neuron in the group of the output neurons with a lower workload of that location, and the neuron with the minimum distance toward the input data. The SOM NN is updated by modifying its weigh vectors $r_{\dot{\alpha }}^{\dot{\eta }}=\langle w_{\dot{\alpha }}^{\dot{\eta }}\left(x\right),w_{\dot{\alpha }}^{\dot{\eta }}\left(y\right)\rangle$ ($\dot{\alpha }=1,2,\cdots ,\alpha$; $\dot{\eta }=1,2,\cdots ,\eta$), by the following rule (17), until the weight vectors remain unchanged.

(17) $$\;\begin{array}{c} r_{\dot{\alpha }}^{\dot{\eta }}\left(t+1\right)\\ =\left\{\begin{array}{cc} G_{i}, & \;\;\mathrm{if}\;D_{\alpha }^{\eta }{|}_{i}<\mu \psi \\ r_{\dot{\alpha }}^{\dot{\eta }}\left(t\right)+\vartheta f\left(d_{\dot{\alpha }}^{\dot{\eta }}\right)\left(G_{i}-r_{\dot{\alpha }}^{\dot{\eta }}\left(t\right)\right), & \;\;\mathrm{otherwise}\; \end{array}\right. \end{array}$$

where ϑ is the learning rate. μ is a little constant, usually smaller than 0.5. ψ is the minimum distance between any two neurons of the goal locations. $f(d_{\dot{\alpha }}^{\dot{\eta }})$ is the neighborhood function, defined as

(18) $$f\left(d_{\dot{\alpha }}^{\dot{\eta }}\right)=\left\{\begin{array}{cc} e^{-{\left(d_{\dot{\alpha }}^{\dot{\eta }}\right)}^{2}/G^{2}}, & \;\mathrm{if}\;d_{\dot{\alpha }}^{\dot{\eta }}<\Lambda \eta \\ 0, & \;\mathrm{otherwise}\; \end{array}\right.$$

where Λ is a small constant denoting the range of neighborhood, normally less than 0.4. $d_{\dot{\alpha }}^{\dot{\eta }}$ is the distance measured between the neuron and the winner neuron, $N_{\dot{\alpha }}^{\dot{\eta }}$, from the group of the output neurons. The gain constant ψ with an initial value of 10 is given by Δ(t+1)=(1−ψ)Δ(t), where ψ is the gain changing rate usually determined empirically. Usually, the range of ψ is between 0.001 and 0.05. Smaller ψ leads to shorter total trajectory and longer computational time. *t* is the number of iterations. The implementation of the proposed multi-robot deployment method is described in Algorithm 2.
**Algorithm 2:** Multi-robot deployment algorithm with distance minimization
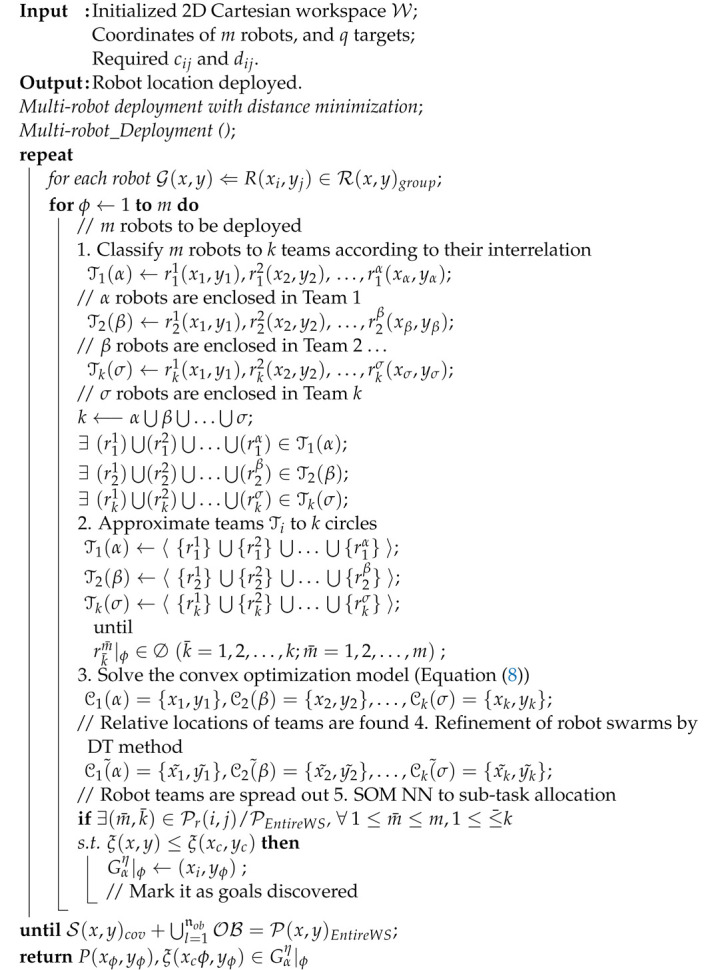


## 5. Simulated Experiments and Comparison Studies

In this section, simulation and comparison studies are conducted to validate our proposed framework.

### 5.1. Numerical Experiments by Convex Optimization

**Figure 8:**
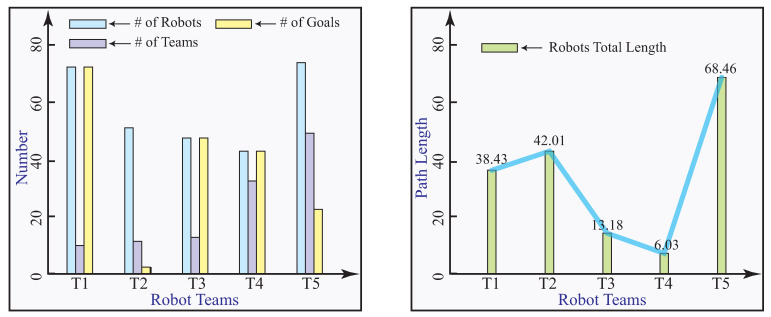
The first set of numerical experimental results.

Numerical experiments are carried out to validate the convex optimization model. In the first set of experiments, we focus on the multi-robot deployment problem through the convex optimization model with equal number of robots and goals, summarized in Table 1. It aims to experimentally verify the correctness of the results obtained from the proposed algorithm. In this experiment, multiple robots are enclosed in their teams before they are deployed to their goals. It assumes that number of robots is equal to the goals, except in Team 2, 50 robots will be assigned to two goals. The total distance the robots traveled is calculated. The first set of experimental results are illustrated in Figure 8.

**Figure 9:**
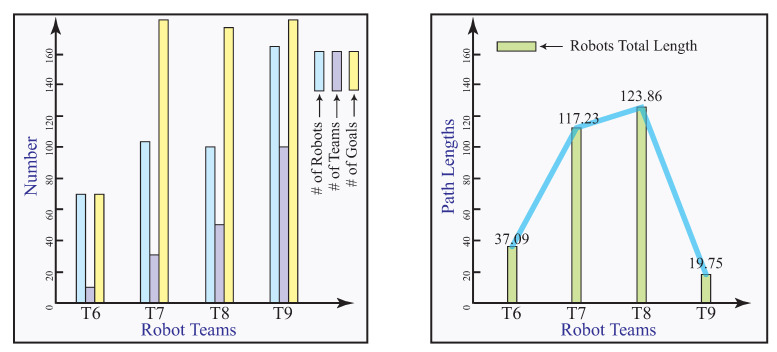
The second set of numerical experimental results.

In the second set of experiments, multi-robot deployment through the proposed convex optimization model is obtained with different numbers of robots and goals. The results are summarized in Table 2. The second set of experimental results are illustrated in Figure 9.

For lack of space, we present our numerical experiments concerning some maps and scenarios, but our results are representative for a broad range of environments. In the next section, we will consider two maps obtained from the publicly available papers.

### 5.2. Evaluation Using Standard Environments

In this section, complex robot allocation experiments in standard environments with multiple constraints are conducted to further validate the robustness and effectiveness of our proposed convex optimization framework. Standard environments, such as apte, xerox, hp, ami33, and ami49, provided by Microelectronics Center of North Carolina (MCNC) are used in our analyses.

**Figure 10:**
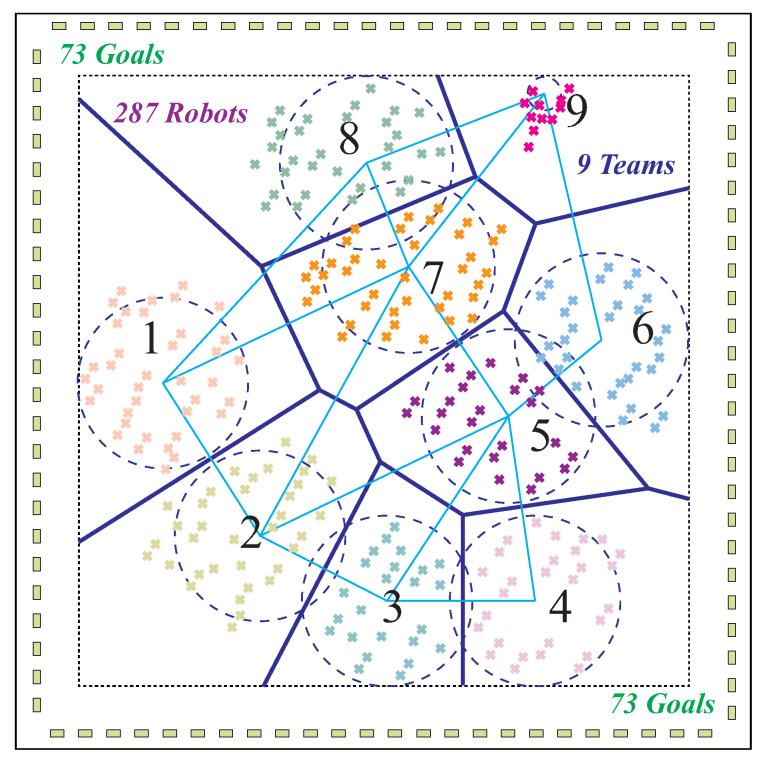
The initial settings of apte standard environment with 287 robots in nine teams. Robots are assigned to goals located on the boundary.

In these scenarios, robots are clustered into predetermined teams. For instance, in the apte environment, 287 robots are distributed into nine teams (Table 3). They are required to perform complex task allocation under 97 constraints. Constraints are limitations or rules that need to be respected by the optimization framework. Several constraints are listed in Table 4. The first constraint is to ensure that nine robots must reach goal location 37. These nine robots must be one from each team $T_{1},T_{2},\ldots ,T_{9}$, respectively, as outlined in Table 4. The second constraint is to ensure that eight robots reach goal location 55, and each of these eight robots must be one from each team $T_{1},T_{2},\ldots ,T_{8}$, respectively. Similarly, the third constraint is that two robots must reach goal location 17, and these two robots must be from groups $T_{1}$ and $T_{2}$. Some robots in the teams are required to reach specific goals. In the apte standard environment, 97 such constraints must be respected. The initial apte environment setting of robots and goals is shown in Figure 10. In this stage, the robots are clustered into nine teams that are assigned to goals located on the boundary. In such scenario, our framework identified an optimized solution on an average in 0.69 s where the average length of all robots path is 425.09 m.

Similarly, in the xerox standard environment with 203 constraints, 698 robots were distributed into 10 teams and were tasked to reach two goals. On average, the optimized solution in this scenario was discovered in 1.12 s where the average path length of all robots is 411 m. Likewise, our framework was able to identify optimal solutions in hp, ami33, and ami49 environments in an average of 1.17, 14.16, and 9.96 s, respectively. As presented in Table 3, the average path lengths for all environments are 154.84, 65.31, and 699 m, respectively. From Table 3, it can also be observed that though number of constraints are more in xerox environment compared to apte, the average length discovered by our framework is smaller in the xerox environment than that of apte. Similar observations can be made from the results for other environments. This is attributed to the fact that not all constraints in different environments are similar. Therefore, the execution time taken by the model and the path length required by the robots to reach, respectively, goals vary significantly based on the environments.

### 5.3. Simulations and Comparison Studies

**Figure 11:**
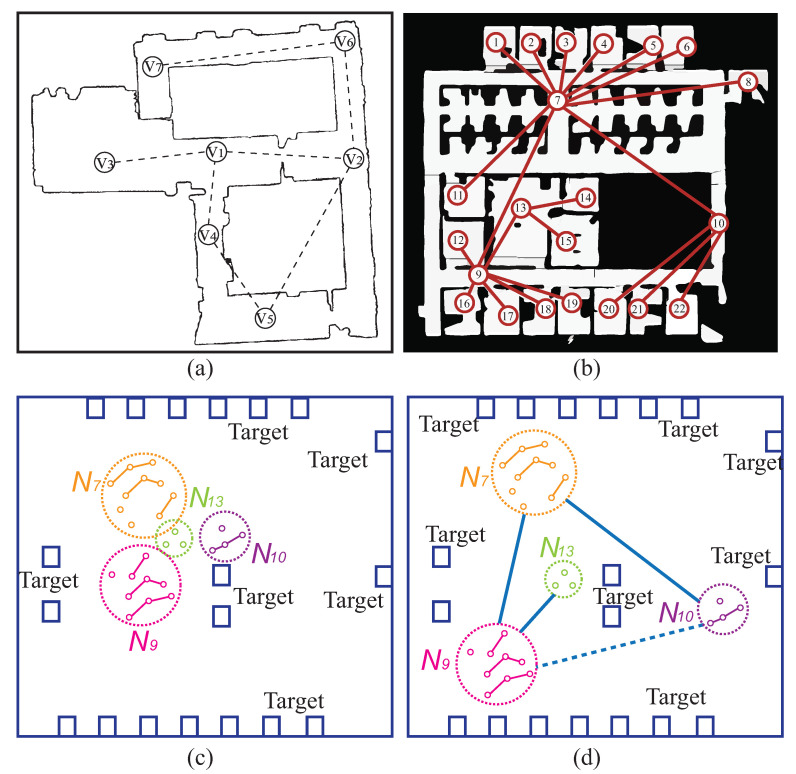
Two SLAM building maps used to test our model. (**a**) Test environment with 7 rooms (redrawn from Purohit et al. [26] Figure 2). (**b**) Test environment with 22 rooms (redrawn from Purohit et al. [26] Figure 3). (**c**) Formation of 4 teams through proposed convex optimization model. (**d**) Spread out teams via Delaunay triangulation (DT).

Two test environmental maps are obtained using a SLAM (Simultaneous Localization and Mapping) algorithm through a publicly available dataset [26]. We used these two building maps to test our framework by assuming the correlative robots using nodes as targets. In this building test scenario, we demonstrate our framework is applicable for the seven-robot deployment. As there are seven nodes corresponding to seven targets, seven robots will be assigned to these seven rooms illustrated in Figure 11a. In this scenario, the deployment aims to deploy at least one robot in each of nodes (rooms). Robots are assigned to seven rooms with minimized travel distance cost, which is formulated to the developed convex optimization model. As this test case is straightforward with only seven targets, the relative locations of solution is obtained from the convex optimization model before they are refined and spread out. Finally, these seven robots are deployed to seven rooms with total distance minimized.

A more complicated indoor room environment is depicted in Figure 11b. In this test scenario, 22 rooms are supposed to be deployed by 22 robots, in which one goal is reached by at least one robot. Our model initially selects Nodes 7, 9, 10, and 13 as the main teams. Based on the interconnection depicted in Figure 11b, a team consists of several robots to be deployed. In this case, Node 7 consists of Robots $r_{1}$, $r_{2}$, $r_{3}$, $r_{4}$,$r_{5}$, $r_{6}$, $r_{11}$, and $r_{9}$. Node 10 contains Robots $r_{20}$, $r_{21}$, and $r_{22}$. Node 13 contains Robots $r_{9}$, $r_{14}$, and $r_{15}$. Node 9 contains Robots $r_{12}$, $r_{16}$, $r_{17}$, and $r_{19}$. Therefore, our developed convex optimization model creates relative locations of Nodes (Teams) 7, 9, 10, and 13. The total distance cost of among these nodes/teams is minimized, where the 22-robot swarms are assigned their final goals in this building.

After applying for the refinement of teams, we obtain their locations of nodes. In each team, it consists of a few robots, such as Node 7 consists of Robots $r_{1}$, $r_{2}$, $r_{3}$, $r_{4}$, $r_{5}$, $r_{6}$, and $r_{11}$. The next stage, these seven robots driven by the SMNN model are navigated to seven rooms. It is clear that node as team containing robots could be the team member of another node. For instance, Node 9 is a member but it is contained in Node 7. This is beneficial of the developed convex optimization able to solve this sort of optimization problem.

In this test scenario of simulation, these 22 robots are clustered into four teams in task decomposition according to their interconnection of robots and correlation of teams. There are four teams, $N_{7}$ with 10 robots; $N_{9}$ with 9 robots; $N_{10}$ with 4 robots; and $N_{13}$ with 3 robots. First stage with our convex optimization model creates relative locations of four teams, in which the total distance cost is minimized shown in Figure 11c. Once the relative locations of these four teams (circles) are determined, a DT method ia applied to spread out them to their appropriate locations shown in Figure 11d. The correlation of teams are shown in Figure 11d. For instance, $N_{7}$ is assumed to connect with $N_{9}$ and $N_{10}$. Robots within their teams are located in vicinity of their targets (rooms). The SOMNN model is used to assign members of robots to their targets (rooms). Therefore, the robots are deployed to their final targets—rooms illustrated in Figure 11b.

**Figure 12:**
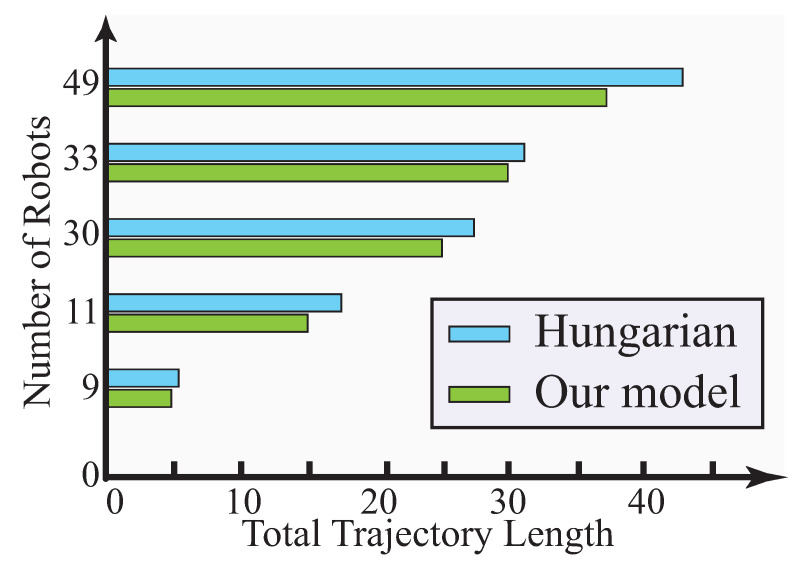
Comparison with Hungarian algorithm in total distance.

Our framework is compared with Hungarian algorithm [51]. Assume that *m* robots are assigned to *k* tasks located at certain goals. The interrelation of robots are assumed. Our framework clusters these robots into teams before they are assigned to goals. Hungarian algorithm directly assigns robots to their tasks and goals. We simulate 6, 11, 30, 33, and 49 robots to be deployed to same number of goals. The results show that, in terms of total path length, our proposed model is 10.14%, 14.26%, 8.69%, 3.96%, and 18.22% lower than the Hungarian algorithm for 6, 11, 30, 33, and 49 targets, respectively. The distance robots traveled to goals as a measure is compared with Hungarian algorithm and illustrated in Figure 12.

## 6. Conclusions

A new framework of team-based multi-robot deployment is proposed through convex optimization model. Multiple robots are deployed to their appropriate locations while the total distance cost is minimized, by the convex optimization mission and robot path planning. The SOMNN paradigm is used to dynamically fulfill the sub-task allocation task. The proposed model couples task decomposition, deployment, local subtask allocation and path planning to support cases where the optimal solution depends on robot interrelation, dependencies, and availability, as well as inter-team conflict avoidance. Simulation and comparison studies validate the effectiveness of our proposed framework. This framework will be implemented on actual robots in the near future work.

## Figures and Tables

**Table 1:** The first set of numerical experiments with robot teams.

Teams	# of Robots	# of Teams	# of Goals	Length
$T_{1}$	73	9	73	38.43
$T_{2}$	50	10	2	42.01
$T_{3}$	45	11	45	13.18
$T_{4}$	42	33	42	6.03
$T_{5}$	75	49	22	68.46

**Table 2:** The second set of numerical experiments with robot teams.

Teams	# of Robots	# of Teams	# of Goals	Length
$T_{6}$	69	10	69	37.09
$T_{7}$	106	30	212	117.23
$T_{8}$	100	50	209	123.86
$T_{9}$	167	100	334	19.75

**Table 3:** The average execution time and path length of the proposed convex optimization-based framework for the standard MCNC environments.

Standard Environment	Goals	Teams	Robots	Constraints	Workspace (m${}^{2}$)	Avg_time (s)	Avg_length (m)
apte	73	9	287	97	46.56	0.69	425.09
xerox	2	10	698	203	19.35	1.12	411
hp	45	11	309	83	8.30	1.17	154.84
ami33	42	33	522	123	1.16	14.16	65.31
ami49	22	49	953	408	35.4	9.96	699

**Table 4:** The constraints in the apte standard environment. 287 robots from nine teams are assigned to 73 goals.

# of Constraints	To # of Goal	# of Robots	From # of Teams
1	37	9	$T_{1}$,$T_{2}$,$T_{3}$,$T_{4}$,$T_{5}$,$T_{6}$,$T_{7}$,$T_{8}$,$T_{9}$
2	55	8	$T_{1}$,$T_{2}$,$T_{3}$,$T_{4}$,$T_{5}$,$T_{6}$,$T_{7}$,$T_{8}$
3	17	2	$T_{1}$,$T_{2}$
4	19	2	$T_{1}$,$T_{2}$
5	16	2	$T_{1}$,$T_{2}$
6	15	2	$T_{1}$,$T_{2}$
7	2	8	$T_{1}$,$T_{2}$,$T_{3}$,$T_{4}$,$T_{5}$,$T_{6}$,$T_{7}$,$T_{8}$
8	14	2	$T_{1}$,$T_{5}$
9	13	2	$T_{1}$,$T_{5}$
…	…	…	…

## Data Availability

Not applicable.
